# Sublethal effects of imidacloprid-contaminated honey stores on colony performance, queens, and worker activities in fall and early winter colonies

**DOI:** 10.1371/journal.pone.0292376

**Published:** 2024-01-02

**Authors:** Mark J. Carroll, Nicholas J. Brown, Dylan Reitz

**Affiliations:** Carl Hayden Bee Research Center USDA-ARS, Tucson, Arizona, United States of America; University of Alberta, CANADA

## Abstract

Neonicotinoid-contaminated sugar stores can have both near term and long term effects on honey bees due to their persistence in honey stores. Effects of imidacloprid food stores contaminants were examined in subtropical colonies that experience reduced brood rearing and foraging during overwintering. Colonies were given treatment sugar syrup containing 0 ppb (control), 20 ppb (field relevant), or 100 ppb (above field relevant) imidacloprid over six weeks to simulate contaminated fall nectar. Colonies were evaluated immediately (post-treatment) and 10 weeks (mid-winter) after treatment to compare proximal and latent effects. Post-treatment 0 ppb and 20 ppb colonies had more workers than 100 ppb colonies while 0 ppb colonies more brood than 20 ppb or 100 ppb colonies. Mid-winter 0 ppb and 20 ppb colonies had more workers than 100 ppb colonies and 0 ppb colonies more brood than 100 ppb colonies. Colonies experienced seasonal declines in stored pollen but no treatment effects. Lower 100 ppb colony performance was associated with reduced effort rather than lifespan. RFID (Radio Frequency Identification) tracking revealed that workers had similar adult lifespans across treatments; however, 100 ppb workers engaged in activities outside the colony for less time than 0 ppb workers. Imidacloprid exposure affected queen but not worker nutritional physiology. Nurses retained well-developed hypopharyngeal glands (as indicated by head protein) across treatments. Mid-winter queens from 0 ppb colonies had marginally higher ovary protein than queens from 100 ppb colonies and more ovary lipids than queens from 20 ppb colonies. However, queen nutrient stores in non-reproductive tissues (fat bodies) did not differ across treatments. Queens from different treatments were attended by comparable numbers of retinue workers and had similar gland contents of four QMP (Queen Mandibular Pheromone) components essential to queen care. High levels of imidacloprid in sugar stores can negatively affect colony performance months after initial storage.

## Introduction

As a species, honey bees (*Apis mellifera* L.) provide key pollination services to crops worldwide and are considered critical pollinators for global food security [1]. Declines among both managed honey bees and native pollinators have been a source of concern worldwide and have been attributed to a combination of stressors including reduced forage, poor nutrition, introduced parasites (Varroa mite) and pathogens including *Nosema ceranae*, and exposure to agrochemicals from cultivated forage environments [2–5]. Among the most common pesticides that honey bees are exposed to are neonicotinoids, neurotoxic synthetic analogs of nicotine [6–7]. Neonicotinoids were first introduced commercially in the 1990s, but are now among the most widely used insecticides worldwide [8–12]. Neonicotinoids are commonly encountered by honey bees in nectar and pollen at chronic sublethal rather than lethal doses due to their persistent systemic characteristics [13–16]. Concerns about the sublethal effects of neonicotinoids stems from the pervasiveness of these insecticides in agricultural and urban environments both as slow-release applications and persistent residues [13, 14, 17–19]. Bee foragers ingest, collect, and come in contact with neonicotinoids in contaminated pollen, nectar, water, guttation fluids, and occasionally, as dust from seed treatments [13, 20, 21]. Effective concentrations encountered by bees vary widely depending on application method and label doses. Most pollens and nectars from treated crops contain less than 10 ppb neonicotinoids; however, some pollens and nectars from specialty crops, ornamentals and field margin weeds have up to 100 ppb neonicotinoids, while guttation water drops and drench application run-off can contain high concentrations of neonicotinoids in the low ppm [13, 21–25, but see 26].

Honey bees encounter a broad range of neonicotinoid concentrations that span the thresholds of most reported sublethal effects [15, 27]. Recent studies have identified a wide array of sublethal effects of neonicotinoids on honey bees, especially in activities involving behavior, learning, and cognition. Sublethal exposure of adult workers to neonicotinoids negatively impacts foraging, navigation, orientation, memory, olfactory learning, brood rearing, waggle dance communication, hypopharyngeal gland (HPG) size, and hygienic behavior [28–38]. Exposures to neonicotinoids depress immune responses, increase susceptibility to *Nosema ceranae*, increase mortality synergistically with pathogens, and increase viral loads in affected workers [39–44]. Queens exposed to high levels of neonicotinoids have reduced oviposition rates, developed fewer ovarioles and experienced lower stored sperm viability, all of which can contribute to lowered queen fecundity [38, 45, 46]. As a result, queens exposed to neonicotinoids may experience higher rates of supersedure, queen loss, and lowered survivorship than unexposed queens [45, 47]. Developing queens reared in colonies exposed to high neonicotinoid concentrations develop smaller pheromone-producing mandibular glands, have lower stored sperm viability, and have lower larval and pupal survivorship [48, 49]. At the colony level, sublethal doses of neonicotinoids may impact colony performance through reduced pollen storage, foraging, brood rearing, and adult worker populations [33, 38]. Neonicotinoids may also act synergistically or antagonistically with other significant stressors such as pathogens and parasites [39, 50, 51]. Many of these sublethal effects have not been observed in honey bees exposed to common field-relevant levels of neonicotinoids (< 10 ppb in pollen and nectar), but rather have been detected in bees exposed to higher residue levels (20 ppb and above) [9, 47 but see 14, 52, 53].

The impact of neonicotinoid-contaminated food stores depends not only on their imidacloprid concentration, but what other food materials are available to colony bees. Neonicotinoid-contaminated honey stores have the potential to affect honey bees not only during immediate collection, storage, and consumption, but also during later consumption of contaminated honey stores by colony members. Unlike water and pollen, sugar is used continuously by a wide variety of colony members including adult workers, older larvae, and drones, and indirectly through glandular trophallactic secretions by queens, younger larvae, and adult bees [54–57]. As pesticides, neonicotinoids break down rapidly (within days) in full sunlight and metabolic processes but degrade slowly in dark environments such as certain soils and stored honey [6, 13, 17, 21, 58, 59]. Honey bees rely extensively on previously-collected honey stores during periods of low nectar availability from the forage landscape. One period of increasing vulnerability to honey contaminants is during fall preparations for colony overwintering when forage is limited. The fall period is especially critical for colony performance in many warm subtropical regions since it represents the last period to collect floral resources and rear large numbers of new workers before the winter dearth [60]. Workers become increasingly dependent on food stores in fall and winter as floral forage and ambient temperatures decrease [61]. In warm winter climates, workers consume large amounts of stored honey both to thermoregulate the hive temperature and to actively scout for forage [62, 63].

We examined both proximal and latent effects of imidacloprid-contaminated sugar sources during periods of high forage availability (mid-fall) and scarcity (winter dearth). Like bees in many subtropical locations, colonies in the Lower Sonoran desert experience a bimodal annual floral forage availability with forage abundance peaking in the spring and fall and pronounced dearths during the mid-summer and early to mid-winter [60]. We gave colonies imidacloprid-contaminated supplemental sugar syrup for six weeks in early fall to simulate exposure to contaminated nectar flows from early fall crops or field weeds [21]. Locally, workers are heavily reliant on honey stores not only during the mid-summer and winter dearths but also during the fall, a period marked by abundant pollen but limited nectar from native floral resources in the landscape [60, 63]. We evaluated colony performance both immediately after supplemental treatment (mid-fall) but also 10 weeks later during the winter dearth when colonies are completely dependent on food stores.

Because high exposures to neonicotinoids are known to impair worker tasks, we also examined effects on individual worker nutrition and maturation factors critical to colony performance. Specifically, young adult workers need to maintain well-developed hypopharyngeal glands (HPG) to produce glandular secretions to feed larvae and other adult bees [56, 57, 64]. Premature loss of HPG and fat bodies among young adult workers is indicative of severe malnutrition or accelerated adult maturation, both signs of stress in themselves. To further examine effects at the individual level, we employed RFID (Radio Frequency Identification) tracking techniques to monitor external hive activities of individual bees. RFID tracking allows for the continuous monitoring of individual workers on a mass scale throughout their adult lives since each RFID tag identifier is unique. RFID tracking has previously been used to examine short term foraging effects as well as long term behavioral changes (i.e. precocious foraging [65]). Here, we extended its application to assess adult worker lifespan and to monitor worker engagement in outside activities including foraging.

We also assessed the effects of exposure to imidacloprid on queen-worker interactions central to colony brood production. In a honey bee colony, a viable ovipositing queen is the only source of female worker offspring used to replenish colony populations [54]. Queens must be fed and attended adequately by workers to support high ovipositing levels in the colony. Queens acquire most of their nutrition from glandular secretions obtained via trophallactic interactions with attendant retinue workers [64]. Conversely, queens project their presence and vitality to the workers in part by gland releases of Queen Mandibular Pheromone (QMP), a five component pheromone complex that is transferred from the queen to the colony by contact with retinue workers [66–70]. These QMP compounds form part of Queen Retinue Pheromone (QRP), a nine component pheromone mixture that elicits retinue care from nest workers [70]. Recent studies have indicated that queens from colonies exposed to high levels of neonicotinoids oviposit slower and may be lost or superseded more often than unexposed colonies [38, 45, 47]. To examine key aspects of worker-queen interactions, we quantified QMP components present in queen mandibular glands as well as the number of retinue workers attending the queen. We also measured the nutrient levels present in queen ovaries and fat bodies as indicators of nutrient provisioning essential for queen oviposition efforts [71].

## Materials and methods

### Honey bee colonies and the forage environment

Honey bee colonies were established from splits of established colonies on 10-frame Langstroth single deep boxes from May to July 2018. At the beginning of the experiment, colonies consisted of approximately 10,000 to 15,000 workers and 5,000 to 8,000 brood headed by a commercially-mated queen introduced that summer. Colonies were kept at an isolated apiary at the University of Arizona Red Rock Agricultural Center (RRAC; Pinal County, AZ, USA; 32.5418–111.3438, elevation 890 m) located in a Lower Sonoran desert creosote (*Larrea divaricata*) shrub community in a moderately-grazed alluvial plain [60]. Forage availability varied seasonally at this site but was minimally available for most of the year except during mid-summer and early winter. Late spring and early summer forage was dominated by mesquite (*Prosopis* spp.), catclaw acacia (*Senegalia greggii*), and various cacti (*Opuntia* spp., *Cylindropuntia* spp. and others). Forage declined during the mid-summer dearth (early July to late August) but increased in the late summer and early fall at RRAC as creosote, mesquite, triangle bursage (*Ambrosia deltoidea*), burroweed (*Ambrosia dumosa*), and various composites bloomed. Forage was briefly unavailable from the first frosts in early December until the first flowering of winter ephemerals and cultivated rapini (*Brassica rapa rapa*) in late December. For managed crops, the RRAC apiary was located 2.7 km from experimental agricultural fields where cotton was grown without pesticide treatment. To limit malnutrition during the mid-summer dearth, colonies were fed supplemental pollen patty (1:1:1 mixture of dried corbicular pollen (Great Lakes Pollen Mix, Great Lakes Bee Supply, Galesburg, MI, USA): Megabee (MegaBee Inc., San Diego, CA, USA): sucrose; 60 g to 220 g pollen patty per week) from June to mid-August. All colonies had abundant sugar stores and most colonies had stored pollen available throughout the experiment.

### Imidacloprid sugar syrup treatments

Honey bee colonies were exposed to imidacloprid in supplemental sugar syrup during the late summer/early fall (9/2/18-10/17/18) to simulate a contaminated nectar flow. Local unmanaged forage in the lower Sonoran desert during this period consists of moderate pollen sources with limited nectar availability [60]. Colony sugar stores were first reduced in late July to approximately 10 to 16 kg of honey frames by the systematic replacement of excess honey frames with empty cell comb frames. Colonies were then equalized twice in late July and mid-August (two weeks before treatment initiation) to roughly equivalent numbers of adult bees, brood, and sugar stores. Fifty-six experimental colonies were then randomly assigned to one of three supplemental sugar syrup treatment groups. During the six-week treatment period, colonies were presented weekly with 3 L 1:1 (w/w sucrose: water) sucrose syrup containing either 0 ppb (control, n = 17), 20 ppb (field-relevant, n = 18), or 100 ppb (above field-relevant, n = 19) (w/w) imidacloprid (Sigma, St. Louis, Missouri, USA) in in-hive top feeders (Brushy Mountain, North Carolina, USA; treatment groups referred to by their concentration hereafter). Imidacloprid-containing syrup solutions were kept out of light at all times since neonicotinoids rapidly degrade in sunlight [18]. Each colony was supplemented over the treatment period with 18 L sucrose syrup, or approximately 9 kg of sucrose, an amount equivalent approximately one third of the average annual surplus honey harvest in commercial colonies in Arizona (National Agricultural Statistics Service, Agricultural Statistics Board, USDA, 2004 to 2011). Bees mainly stored the supplemental sugar syrup in frame cells as evidenced by the removal of all syrup and short term gains in colony mass.

Each colony’s resident queen was marked to distinguish her from daughter queens or Africanized invasion queens [72]. Colonies were examined monthly for the presence and oviposition activity of the resident queen (as evidenced by brood production). Queen events (i.e. queen cells, virgin queens, supersedure/invasion queens, absence of marked queen) were noted and cells removed. Colonies that lost a marked queen were not requeened and were allowed to requeen or fail naturally. Colonies that naturally requeened were excluded further from the experiment due to the likely subsequent production of Africanized or hybrid workers by the new queen. Colonies were treated for Varroa mites during June 2018 with three applications of Apiguard (thymol, Vita Europe Ltd., Basingstoke, UK). Phoretic mite infestation rates among nest workers were periodically estimated from nest workers by alcohol wash, but did not exceed 3% during the experiment in any colony.

### Colony agrochemical residues

Adult bees, stored pollen and honey stores were sampled from colonies at each time point (see below) to assess exposures to colony pesticides from both treatment and external sources during the experiment. Approximately 20 adult nest bee workers were sampled from the center of the colony and about 1 g stored pollen and 1 g capped sugar stores were sampled from at least 12 locations in each colony. Subsamples from all surviving colonies in each treatment group were pooled equally to provide approximately 3 g material for a full pesticide panel analysis from the USDA Agricultural Marketing Service National Science Laboratory in Gastonia, NC, USA.

### Colony performance evaluations and sampling

Colonies were sampled and evaluated at three time points before and after the six week syrup treatment period (sugar syrup treatment 9/2/18-10/17/18; evaluations for pre-treatment 8/24/18-9/1/18, post-treatment 10/18/18-10/21/18, and mid-winter (10 weeks after treatment) 12/24/18-12/25/18)). To limit stress on queen retinues (see below), colony evaluations were only conducted after the queen and workers were removed for retinue behavior observations. All evaluations were performed in warm daytime conditions (25°C to 28°C) and completed within 8 minutes to limit effects on brood. During the first two time points, each queen’s interactions with retinue workers were recorded, while during the last time point, the queen was sampled for analyses of queen pheromone glandular contents, stored sperm (in spermatheca), and internal nutrient contents (see below). At each time point, nurse bees were sampled from the brood center to provide estimates of worker nutritional health (see below) and estimates were made of colony performance (adult worker population, brood population, and stored pollen).

The total adult worker population was estimated for each colony at each time point by calculating the total mass of adult workers present in the colony [73]. Each colony was weighed intact before dawn (before worker flight) to obtain a colony mass with all adult workers present. Later that day, colony brood and hive components were weighed without adult workers. These measurements were conducted carefully but rapidly (within 6–8 minutes) to limit brood stress from exposure and brood neglect. The queen was located and caged and workers were either carefully shaken off or brushed off the frames into a holding box. The remaining frames and hive equipment were then weighed to obtain a colony mass without the adult worker component. Brood and stored pollen frame photographs were then taken of frames cleared of workers (see below), the colony rapidly reassembled, and the queen and workers returned to the brood nest core.

The total number of brood and stored pollen cells were estimated by analysis of frame photographs using ImageJ version 1.47 software (W. Rasband, National Institutes of Health, USA) [63]. Photographs of each frame containing brood or stored pollen were taken on a frame holder rig that fixed the distance (0.72 m) and angle (perpendicular) between the camera and the center of the frame face. The frame rig was oriented relative to the sun so that sunlight illuminated most of the cell contents. Brood and pollen cell patches were outlined using the software and converted to comb area using the inner perimeter of the wooden frame as a known fixed reference distance. Isolated cells outside the outline were manually counted. Software-estimated cell area was converted to cell number using known values for cell numbers per unit comb area.

### RFID tracking of adult worker longevity and activity outside the colony

Young adult workers of known age were tagged and tracked by RFID to assess imidacloprid treatment effects on worker longevity and adult lifetime activities outside the colony (including foraging) during the critical fall (pre-winter) period [65]. The activity of individual workers could be linked to specific colonies because each RFID tag and colony reader/detector carries an unique identification code (Microsensys Inc., Erfurt, Germany). Young adult workers were tagged and tracked starting approximately 4 weeks into the syrup treatment (10/3/18 to 10/6/18) to ensure that these workers and their older caretakers had been exposed to treatment syrups throughout the young worker’s life. Five colonies were selected from each treatment group for RFID monitoring of their hive entrance. Selected workers of known age were tagged 3 days after adult emergence not only to avoid injury to newly emerged workers but also to tag the workers before their first outdoor flights. Approximately 100 newly emerged adult workers per colony were paint marked by colony with a unique color and carefully returned to each colony. At 3 days post-emergence, marked workers had a Microsensys mic-III tag (Microsensys Inc., Erfurt, Germany) affixed to their dorsal thorax with Loctite Super Glue (Westlake, OH, USA). Freshly-tagged workers (50 per colony except one erroneously given 60) were caged on colony comb for a few hours before re-entry to limit rejection by other colony workers. Tagged worker activities outside the colony were detected by the placement of a Microsensys MAJA III System reader with MAJA III data recorder and data connector box across the colony entrance. Colonies were otherwise sealed with tape to avoid exits through unmonitored gaps. Colonies were monitored for 33 days after RFID tagging (37 days after adult emergence, long after normal age-of-first foraging) in order to detect longer-lived diutinus workers. Tag reads were collected by the MAJA III data box and exported as a raw data Excel file for analysis. UTC timestamps were converted to decimal days with only reads between 4 am and 9 pm considered as potential trips.

RFID was used to estimate the total time spent outside the colony (a proxy for foraging and other outside activities) and the last day an individual was detected (an approximation of minimum adult worker lifespan). RFID readers were capable of detecting the presence of a bee under the entrance reader but not the motion or direction of worker movements. The total time spent outside the colony was calculated for each individual as the time between a pair of successive reads (i.e. a trip). An even number of reads was assumed to constitute a series of round trips while an odd number indicated a bee that either died outside, remained outside of the colony for an extended period (including daytime and overnight “bearding”), was removed outside the colony, or joined an unmonitored colony. Alternatively, an odd number of reads could result from a tag that was not read, a read repeated multiple times in one pass, or a tag that fell off. Only multiple read tagged workers (55.3% (99/179), 50.2% (112/223), and 55.4% (123/222) of detected workers in the 0 ppb, 20 ppb, and 100 ppb treatment groups respectively) were compared. Undetected and single read tagged workers were eliminated from the analysis. Colony workers rapidly removed dead or injured bees from the hive throughout the monitoring period so removal was assumed to approximate earliest possible time of death. To eliminate multiple reads from stationary workers positioned below the reader, all successive reads from a given tag less than 1 minute in separation were manually consolidated into one read. Odd reads were corrected by pairwise averaging of all possible time points (assuming one was missing or extra) using an R script program in part to eliminate extraneous or multiple reads (script by William Meikle using R Foundation for Statistical Computing, www.r-project.org). Each worker’s outside activity was estimated by summation of the total trip times for each individual worker. Multiple read tags with a calculated outside activity time of less than 1 minute (36) were assumed be equivalent to a single read (one way removal of the tag) and eliminated from the analysis. Not all the time spent outside was spent foraging or engaged in active flying–individuals could accumulate considerable time positioned on the external surfaces of the colony or by residing in an unmonitored colony. The minimum adult worker longevity was estimated from the last detected read of a multiple read tagged worker (i.e. removal of a dead/injured worker or last time the tagged worker left the colony). This metric was capped by the maximum duration of RFID monitoring (37 days after adult emergence/33 days after RFID monitoring started) but allowed for detection of longer lived workers.

### Individual nest worker head protein contents

The nutrient state of colony workers was estimated at each time point by quantifying nutrients from worker tissues associated with brood rearing and behavioral maturation [74, 75]. To detect severe malnutrition among food providers, we assessed the soluble protein present in the worker head, a structure that consists partially of hypopharyngeal glands (HPG) used to feed larvae, queens and other workers [76]. Five nest workers actively engaged in nursing larvae were randomly sampled from the brood center of each colony. HPG development was indirectly quantified as the total soluble protein present in dissected worker heads by a bicinchonic acid (BCA) protein assay (Pierce Thomas Scientific, MA, USA) [73]. At each colony time point, a pooled sample of five worker heads was homogenized in 1 mL PBS buffer by a Bead Beater (3 x 30 sec, BioSpec Products, Bartlesville, OK, USA), and centrifuged. Two hundred μL supernatant was diluted in 800 μL PBS buffer and centrifuged. The diluted homogenate was then analyzed for total soluble head protein by a bicinchoninic acid (BCA) assay (Pierce BCA Protein Assay kit, Thermo Fisher Scientific, Waltham, MA, USA). Twenty five μL homogenate was reacted with 175 μL BCA reactant solution in a 96 well plate at 32°C. Total soluble protein was estimated by comparing sample 562 nm absorbance against a bovine saline albumin (BSA; Sigma Inc., St. Louis, MO, USA) standard curve in a Gen-5 Plate Reader (Biotek, Inc., Winooski, UT, USA).

### Queen interactions with retinue workers

The effect of imidacloprid on worker attendance of queens was assessed during the pre-treatment and post-treatment evaluations by quantifying the number of retinue workers attending the queen [66, 68–70, 77]. Retinue interactions between the resident queen and at least 120 nest workers were video-recorded *in situ* on an observation frame over a 10 minute period. Observations were conducted only with undisturbed queens and workers from the brood center and colony entries were made without the use of smoke. All observations were conducted in a shaded tent at ambient temperatures between 26°C to 31°C to minimize disturbance. Disturbed queens and/or workers were returned to the colony brood center and observed on a different day. Once the queen was located on a brood frame, a metal observation frame was pushed into the inner perimeter of the brood frame to encourage the queen and workers to remain on that frame face. The enclosed frame was mounted on a frame holder and queen-worker interactions were recorded by a Sony HandiCam camcorder (mounted on a tripod with a 16 or 32 MB data card). The largest worker retinue observed within each one minute interval from the video recordings was enumerated. Workers were rated as retinue workers if they were 1) touching or licking the queen, 2) oriented toward the queen for 10 seconds, or 3) stationary or nearly stationary facing the queen for 10 seconds (after Seeley 1979 [68]). At each evaluation time point, queens were compared based on the average size of their top three retinue formations in the 10 minute session.

### Queen dissections and spermatheca sperm contents

Mid-winter queens were analyzed to provide estimates of spermatheca sperm contents, queen pheromone (QMP) gland contents, and nutrient contents of reproductive and non-reproductive tissues. The queen head, fat bodies (attached to the abdominal carcass tergites and sternites), ovaries, and spermatheca were removed and re-frozen for separate analyses. The number of sperm present in the queen spermatheca was estimated using hemocytometer counts [78]. The queen spermatheca was dissected into 950 μL HEPES pH 7.4 buffer and 50 μL 10% Bovine Serum Albumin (BSA; Sigma, St. Louis, MO, USA) and gently vortexed. The resulting sperm suspension, which consisted entirely of dead (frozen) sperm, was stained with 5 μL 2.4 mM propidium iodide (Live/Dead Sperm Viability kit, Thermo Fisher Scientific, Waltham, MA, USA). One μL stained suspension was visualized on a hemocytometer by fluorescence microscopy (Nikon Eclipse 80i microscope with D-FL Epi Fluorescence attachment, Nikon Instruments, Melville, NY, USA) at a fluorescence emission of 617 nm.

### QMP contents of queen mandibular glands

The amount of QMP (Queen Mandibular Pheromone) components were quantified in queen mandibular glands of mid-winter queens as a proxy for pheromone release rates to workers [66, 67]. Deep frozen queen heads were placed into 10 mL diethyl ether that contained 60 μg cis-10-heptadecanoic acid internal standard, macerated by iridectomy scissors and extracted at 25°C for 24 hours in the dark [after 79]. Precisely 1.000 mL of the ether supernatant was transferred into a crimp vial, dried down, and silylated with 150 μL BSA (N,O-bis(trimethylsilyl)acetamide, Sigma, St. Louis, MO, USA) at 25°C for 16 hours in the dark. Approximately 100 μL of the silylated extract was centrifuged to pellet fine particulates, removed to a 200 μL vial glass insert, and analyzed by GC-MS. Silylated compounds were separated by EI GC-MS on an HP 7890A gas chromatograph coupled to an HP 5975D mass spectrometer detector. Exactly 1.0 μL sample solution was injected at 220°C onto an Agilent HP-5MS column (30 m x 0.250 mm ID x 0.25 μm film; Agilent Technologies, Santa Clara, CA, USA) at a flow rate of 1.2 mL/min. Compounds were separated by oven temperatures programmed from 40°C (0.5 min initial hold) to 220°C (15 min final hold) at 15°C/min. QMP compounds were identified by comparison of mass spectra and retention times with known standards (Sigma, St. Louis, MO, USA; Acros, Pittsburgh, PA, USA; gift of Contech Enterprises, Inc., Delta, BC, Canada). Five major QMP compounds were identified (methyl-p-hydroxybenzoate (HOB), 4-hydroxy-3-methoxyphenylethanol (HVA), 9-hydroxy-2-decenoic acid (9-HDA), and 9-oxo-2-decenoic acid (9-ODA)) but reported as four since the two enatiomers of 9-HDA were largely indistinguishable. Glandular amounts were reported as relative amounts (peak area counts) rather than absolute amounts for each compound. Peak areas counts of characteristic m/z fragments were obtained in SIM mode with Agilent Chemstation (Agilent Technologies, Santa Clara, CA, USA). We estimated the fraction of the total sample present in the injection from the amount of internal standard recovered in the injected sample.

### Queen ovary and fat body soluble protein and lipid contents

Nutrient contents of queen ovaries and fat bodies were quantified from each mid-winter queen to examine nutrient partitioning between reproductive and non-reproductive tissues [71]. Each tissue was placed in 500 μL PBS buffer and homogenized for 30 sec by Bead Beater with 1.0 mm zirconium beads (BioSpec Products, Bartlesville, OK, USA). For total protein contents, 100 uL homogenate was first diluted in 400 uL PBS buffer. Fifty uL of the diluted homogenate was further diluted in 450 uL PBS buffer. Twenty five uL diluted homogenate was then analyzed for total soluble protein by a BCA assay (Pierce BCA Protein Assay kit, Thermo Fisher Scientific, Waltham, MA, USA) as described above.

Total lipid contents were quantified for each tissue by a modified chromic acid assay on Folch extracts [80]. An 84 μL homogenate subsample was added to 1000 μL 2:1 chloroform: methanol and vortexed for 10 sec. 126 uL 0.25% KCl was added, vortexed, and centrifuged to obtain a partitioned Folch extract. Eighty μL of the lower chloroform: methanol layer was transferred to a crimp seal vial, reduced to dryness, and reacted with 1.000 mL chromic acid at 95°C for 1h. Total lipid content was quantified by comparing the 620 nm absorbance of sample reactant solution against a reacted oleic acid (Sigma Inc., St. Louis, MO, USA) standard curve in a Gen-5 Plate Reader.

### Statistical analyses

All comparisons were made at the colony level across imidacloprid treatment groups. Colony survivorship/mortality at the mid-winter experimental endpoint was compared by Pearson’s Chi square test of independence [81]. The remaining statistical analyses were performed with SAS 9.4 (SAS, Inc., Cary, NC, USA). All other analyses were either performed on colonies/pooled samples repeatedly sampled over successive time points or bees sampled/monitored at one time point. Comparisons were only made on colonies that survived the full duration (two time points for colony performance (adult worker mass, brood populations, stored pollen cells, three time points for worker HPG protein contents) of the experiment. Data sets were checked for normality by Shapiro-Wilks tests and by examination of residuals (PROC UNIVARIATE). Colony brood cells, colony stored pollen cells, worker head protein contents, and queen retinue sizes were each compared by repeated measures analysis (PROC MIXED). Only metrics for queens that survived through the experiment were compared by repeated measures. Data sets with normality (queen spermatheca sperm counts, queen ovary protein and lipid contents, and queen fat body protein and lipid contents) were compared by one-way ANOVA (PROC GLM). For significant one-way ANOVA, multiple comparisons were made by Tukey’s HSD test on individual/pooled colony means. Data sets that lacked normality (total colony adult worker mass (population), RFID minimum worker longevity (last day detected), RFID time workers spent outside the colony) were compared by Kruskal-Wallis tests (NPAR1WAY) conducted separately at each time point. For significant Kruskal-Wallis tests, multiple comparisons of treatment groups were made by performing Dwass, Steel, Critchlow-Fligner (DSCF) tests on colony ranks (PROC NPAR1WAY option dscf). Four queen QMP compounds from queen heads were first compared by PCA analysis (PROC PRINCOMP) to reduce the four initial variables to two principal components (PC1 and PC2) on a scatterplot. Each principal component was compared across treatments by one-way ANOVA (PROC GLM). Each individual QMP component was also compared separately across treatments by a Kruskal-Wallis test (NPAR1WAY) with DSCF multiple comparisons.

## Results

### Colony survivorship

Colonies experienced similar survivorship from late summer to mid-winter across treatment groups with 84% (16/19) of 0 ppb, 83% (15/18) of 20 ppb, and 82% (14/17) of 100 ppb colonies surviving this period (Pearson Chi-square test of independence, Χ^2^ = 0.0224, df = 2, p = 0.99). The majority of colony failures (89%) occurred between the post-treatment and mid-winter evaluations. Three very small colonies (less than 1,000 workers, one from each treatment group) were being actively robbed of honey during the mid-winter evaluations and failed within the week. All failed colonies contained honey and most contained pollen stores. Most colonies that failed during the post-treatment to mid-winter interval (mid-October to late December) experienced sharp reductions in adult populations without sufficient brood rearing to replace lost workers.

### Colony agrochemical residues

Agrochemical contamination of stored pollen and honey was limited to the miticide thymol, an insecticide and two fungicides. Nest bees had trace amounts of sulfoxaflor and large amounts of thymol (897 ppb) used as a miticide during the summer. Honey stores collected before the treatment had minor amounts of carbendazim (11 ppb), Amitraz-related 2,4-dimethylphenylformamide (DMPF; 74 ppb), diuron (1 ppb), thymol (52 ppb), and trace amounts of sulfoxaflor while stored pollen contained sulfoxaflor (12 ppb), tebuconazole (29 ppb), thymol (22 ppb), and trace amounts of carbendazim. Honey stores collected during the syrup treatment period had 6 ppb, 28 ppb, and 111 ppb imidacloprid for the 0 ppb, 20 ppb, and 100 ppb treatment groups while stored pollen contained 0 ppb, 0 ppb, and 25 ppb imidacloprid. Individual workers were heavily contaminated and contained 15 ppb, 29 ppb, and 107 ppb imidacloprid in their bodies and 14 ppb, 54 ppb, and 287 ppb imidacloprid in their heads for the 0 ppb, 20 ppb, and 100 ppb treatment groups. Imidacloprid residues in bodies were present both in honey stomach contents and dispersed throughout their bodies [58].

### Colony performance

**Fig 1:**
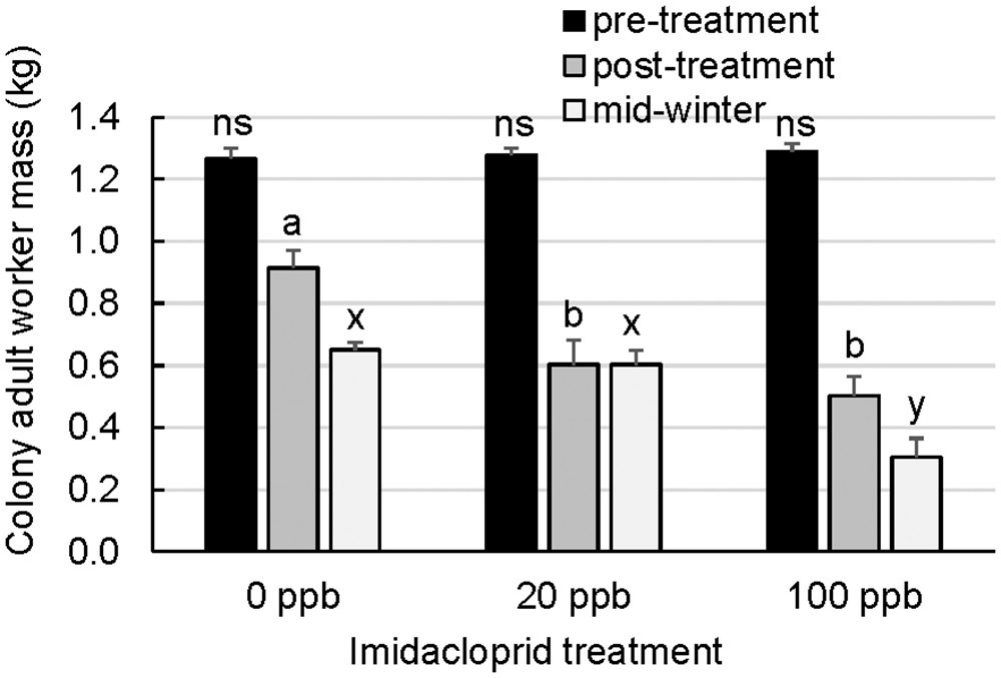
Total adult worker mass of imidacloprid-supplemented and unsupplemented colonies during pre-treatment, post-treatment, and mid-winter evaluations. The adult population sizes of colonies supplemented with 0, 20, or 100 ppb imidacloprid sugar syrup during the treatment phase were estimated by mass at the pre-treatment (August), post-treatment (October), and mid-winter (December) evaluation time points. Error bars are S.E. (colonies n = 16 (0 ppb), n = 15 (20 ppb), or n = 4 (100 ppb, first to last time point)). For a given time point, treatment groups with different letters are significantly different by DSCF (colony ranks).

**Fig 2:**
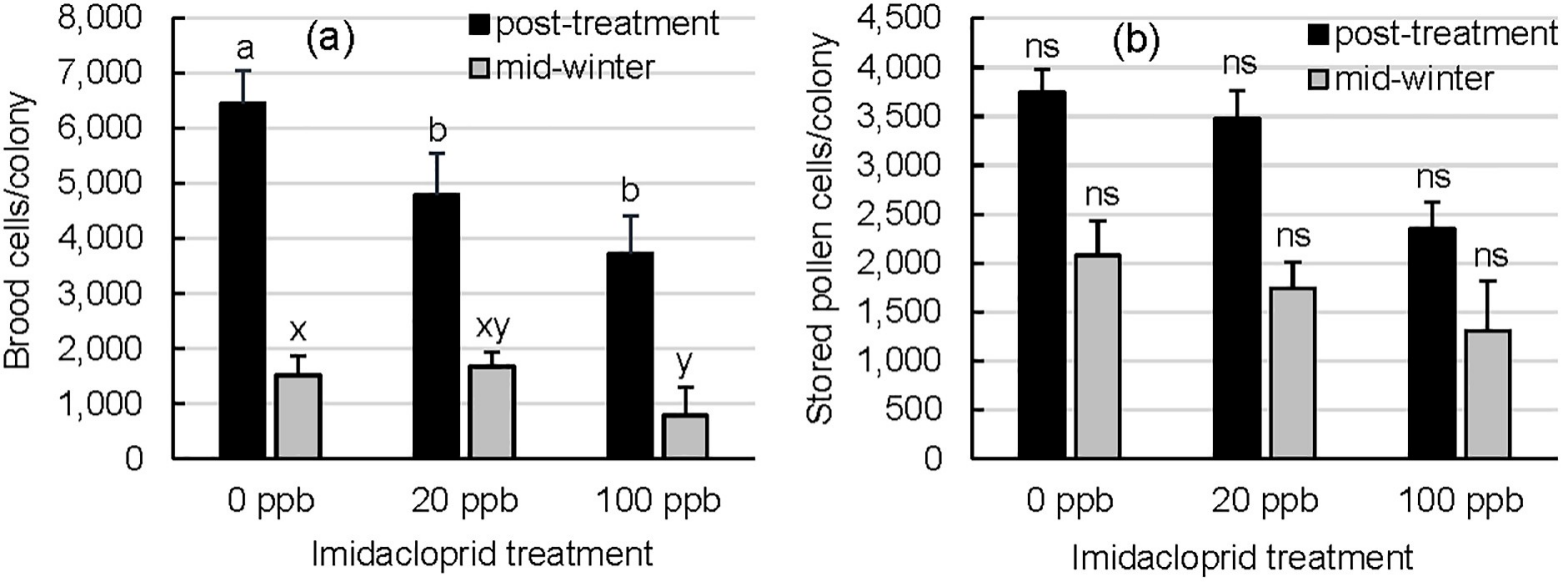
a) Brood and b) stored pollen cells in imidacloprid-supplemented and unsupplemented colonies during post-treatment and mid-winter evaluations. Error bars are S.E. (n = 15 (0 ppb imidacloprid in sugar syrup), n = 12 (20 ppb), or n = 13 (100 ppb)). For a given time point, treatment groups with different letters are significantly different by Tukey’s HSD (colony means).

Colonies experienced seasonal declines from late summer to mid-winter in adult worker populations (estimated by mass), brood populations, and stored pollen irrespective of treatments (Figs 1, 2a and 2b). However, imidacloprid treatments had a marked effect on adult worker populations both immediately after cessation of syrup treatments (post-treatment) and later during mid-winter (Fig 1; Kruskal-Wallis test, Χ^2^ = 0.455, df = 2, p = 0.7967 (pre-treatment); Kruskal-Wallis test, Χ^2^ = 20.803, df = 2, p<0.0001 (post-treatment); Kruskal-Wallis test, Χ^2^ = 17.054, df = 2, p = 0.0002 (mid-winter)). Post-treatment adult worker populations in 0 ppb colonies were significantly larger than 20 ppb or 100 ppb colonies, while mid-winter 0 ppb and 20 ppb colonies had larger adult populations than 100 ppb colonies (DSCF (colony ranks), p<0.05). On average, mid-winter 100 ppb colonies had 47% and 52% of the adult worker populations observed in 0 ppb or 20 ppb colonies.

Neonicotinoid-exposed colonies experienced sharp reductions in brood cell numbers during the critical fall brood rearing period (Fig 2a; repeated measures ANOVA (PROC MIXED) F_2,37_ = 5.70, p = 0.0069 (treatment), F_1,37_ = 91.12, p<0.0001 (time point), F_2,37_ = 2.53, p = 0.0937 (treatment*time point)). At the post-treatment evaluation, 0 ppb colonies reared more brood than 20 ppb or 100 ppb colonies (Tukey’s HSD (colony means), p<0.05). By contrast, mid-winter 0 ppb colonies reared more brood than 100 ppb colonies, but not 20 ppb colonies (Tukey’s HSD (colony means), p>0.05). However, mid-winter brood estimates occurred during the winter brood rearing hiatus. Winter brood counts may have been artificially inflated by our inability to distinguish between live capped brood and dead capped brood not fully removed by workers from weak colonies in the frame photographs.

Colonies displayed seasonal declines in stored pollen cell areas from mid-fall (post-treatment) to mid-winter; however, imidacloprid treatments did not have an effect (Fig 2b; repeated measures ANOVA (PROC MIXED) F_2,37_ = 1.77, p = 0.1837 (treatment), F_1,37_ = 16.44, p = 0.0002 (time point), F_2,37_ = 0.17, p = 0.8444 (treatment*time point)). Stored pollen was available in colonies through most of the experiment, although four colonies completely depleted their pollen stores by mid-winter.

### RFID tracking of adult worker longevity and outside activity (including foraging)

**Fig 3:**
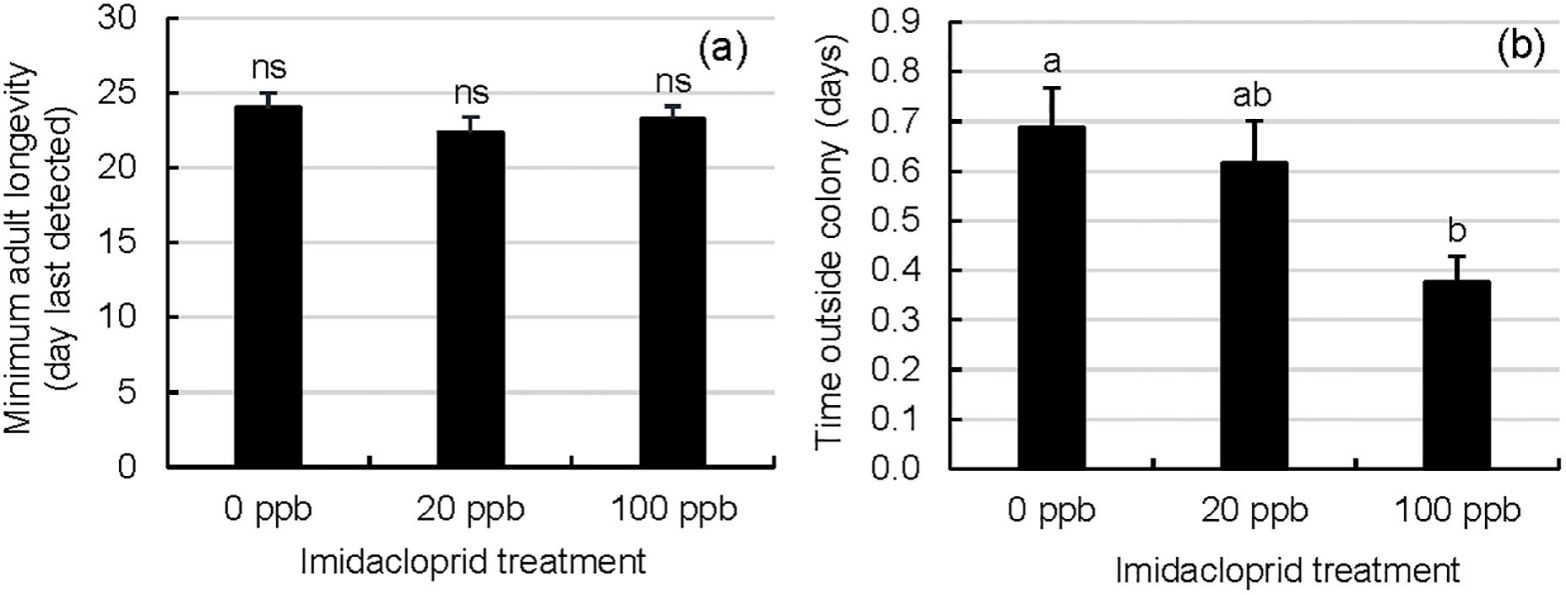
RFID monitoring of worker activities outside the colony in tagged workers from imidacloprid-supplemented and unsupplemented colonies. RFID-tagged workers were monitored for a) their minimum adult longevity (last day detected) and b) the amount of time they spent outside the colony for up to 37 days after adult emergence. Error bars are S.E. (monitored workers n = 99 (0 ppb imidacloprid in sugar syrup), n = 112 (20 ppb), or n = 123(100 ppb)). For a given metric, treatment groups with different letters are significantly different by DSCF (colony ranks).

Exposure to imidacloprid-contaminated syrup affected the amount of time workers engaged in outside activities but not adult worker lifespan. The minimum adult worker lifespan, as estimated by each worker’s last detected read, was relatively short and did not vary significantly among treatment groups (Fig 3a; Kruskal-Wallis test, Χ^2^ = 1.2042, df = 2, p = 0.5477). Lifespan estimates were artificially limited by the RFID monitoring duration (37 days after adult emergence) which did not extend to observed months-long lifespans of diatinus workers; however, 24.9% (83/334) of tagged multi-read workers were detected during the last 5 days of monitoring, a figure that indicated the presence of long-lived workers. By contrast, workers varied significantly in their activities outside the colony (as measured by the total time spent outside the colony) (Fig 3b; Kruskal-Wallis test, Χ^2^ = 7.9110, df = 2, p = 0.0191). Tagged workers from 100 ppb colonies spent less time outside the colony than tagged workers from 0 ppb, but not 20 ppb colonies (DSCF (colony ranks), p<0.05).

### Individual nurse worker head protein contents

To determine if reductions in colony populations and outside activities affected colony nutrition, the nutrient stores of individual nest workers actively engaged in nursing larvae were quantified. Soluble head protein contents (a proxy for HPG) remained marginally similar in nurse workers across treatments, but partially changed over time (S1 Fig; repeated measures ANOVA (PROC MIXED) F_2,45_ = 2.88, p = 0.0677 (treatment), F_1,45_ = 7.56, p = 0.0015 (time point), F_2,45_ = 0.64, p = 0.6390 (treatment*time point)).

### Queen interactions with retinue workers

**Fig 4:**
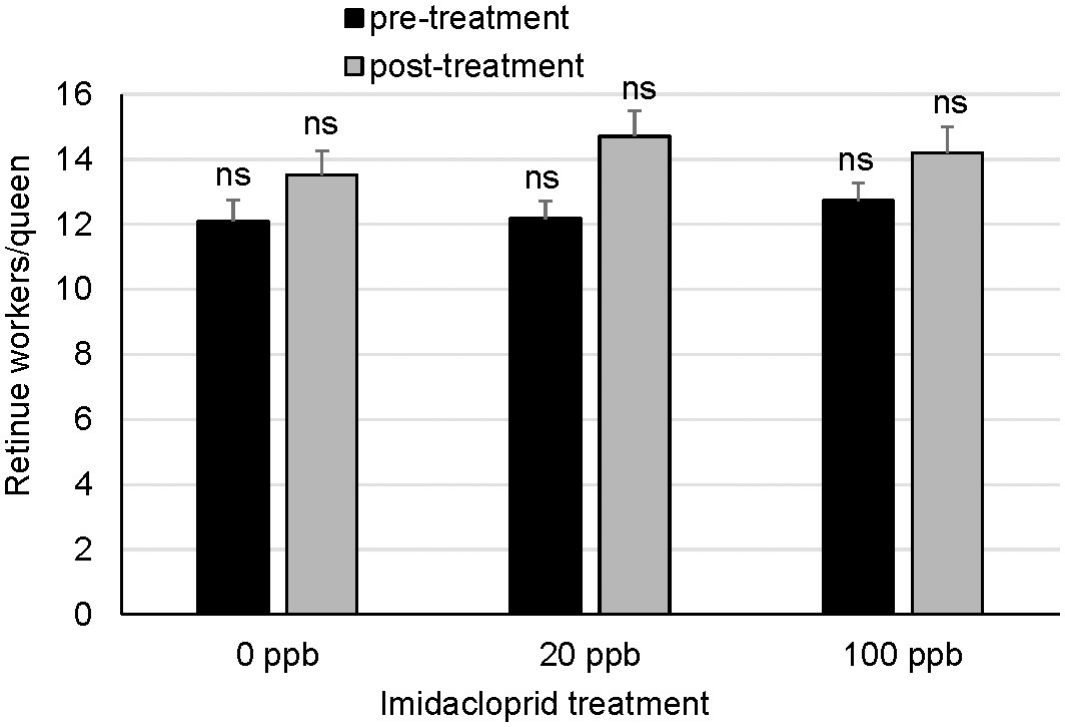
Retinue workers attending queens from imidacloprid-supplemented and unsupplemented colonies during the pre-treatment and post-treatment evaluations. Error bars are S.E. (retinues n = 13 (pre-treatment and post-treatment; 0 ppb imidacloprid in sugar syrup), n = 13 (20 ppb), or n = 14 (100 ppb)). Treatment groups were not significantly different within each time point by Tukey’s HSD (retinue means).

Queens were attended by more retinue workers during the post-treatment than the pre-treatment observations but did not differ across treatments (Fig 4; repeated measures ANOVA (PROC MIXED) F_2,37_ = 1.77, p = 0.1840 (treatment), F_1,37_ = 7.43, p = 0.0097 (time point), F_2,37_ = 1.44, p = 0.2505 (treatment*time point). The post-treatment retinue observations were made during a period of pollen forage availability.

### Queen spermatheca sperm counts

Imidacloprid treatment did not affect the amount of sperm stored in queen spermatheca, despite nearly four months of exposure to treatment syrups (S2 Fig; one-way ANOVA (PROC GLM), F_2,34_ = 0.26, p = 0.7756). Similarly mated queens were observed in colonies across time points and treatment groups despite sharp differences in colony brood rearing, especially during the mid-winter dearth. Among sampled mid-winter queens, almost all queens contained adequate levels of stored sperm for continued oviposition. Two of 37 queens examined were completely depleted of stored sperm in their spermatheca.

### QMP contents of queen mandibular glands

**Fig 5:**
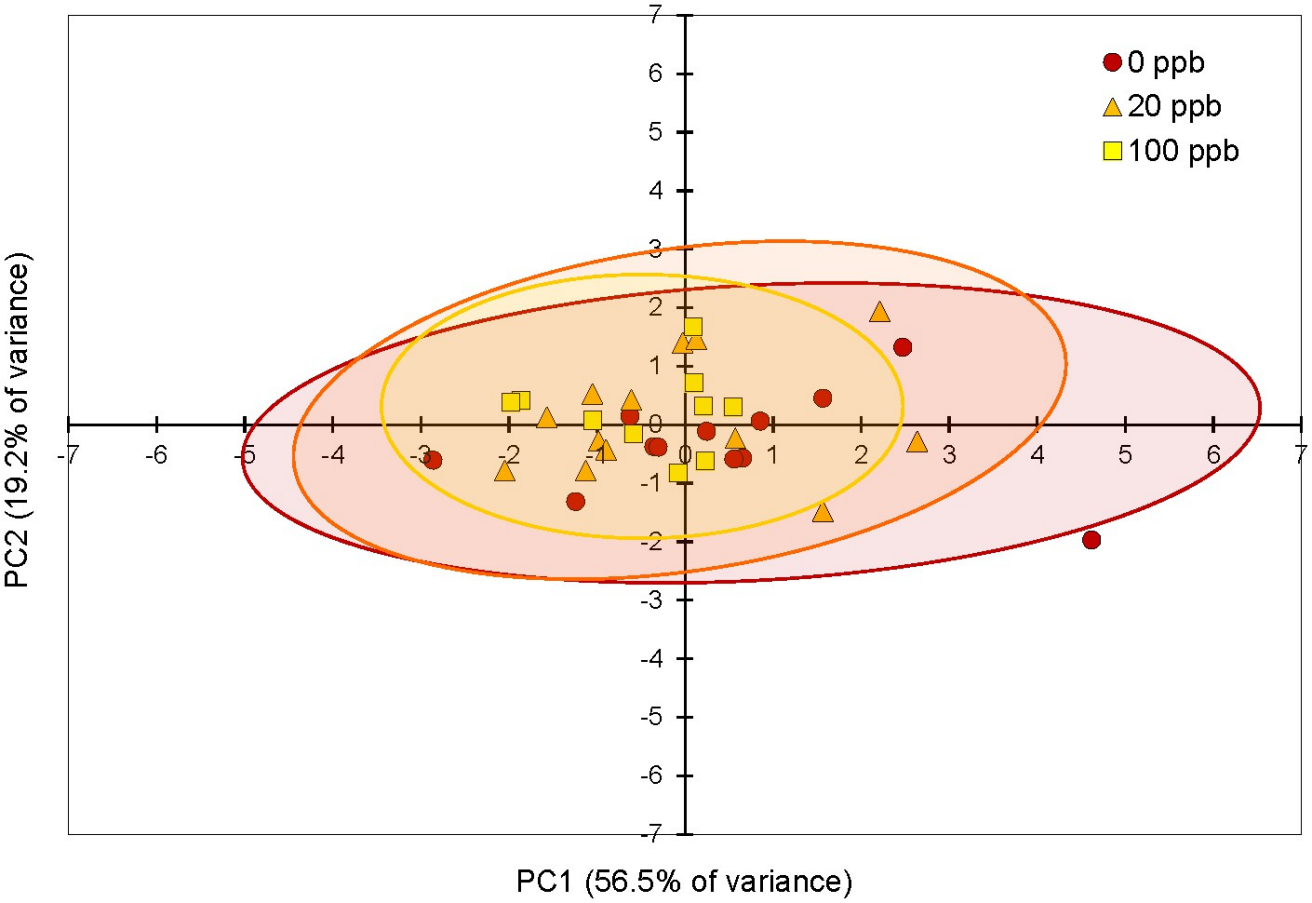
PCA analysis of QMP components isolated from winter queens from imidacloprid-supplemented and unsupplemented colonies. The Queen Mandibular Pheromone (QMP) components methyl-p-hydroxybenzoate (HOB), 4-hydroxy-3-methoxyphenylethanol (HVA), 9-hydroxy-2-decenoic acid (9-HDA) and 9-oxo-2-decenoic acid (9-ODA)) were extracted from queen mandibular glands. The two enantiomers of 9-HDA are reported together since these were largely indistinguishable. PC1 explained 56.5% and PC2 explained 19.2% of the variance between groups. These principal components did not differ significantly between treatment groups (one-way ANOVA, (PROC ANOVA), F_2,32_ = 1.02, p = 0.3706 (PC1); F_2,32_ = 1.33, p = 0.2785 (PC2)). Color-coded ellipses indicate 95% confidence levels for PC1 and PC2 on the scatterplot for each indicated treatment group.

**Fig 6:**
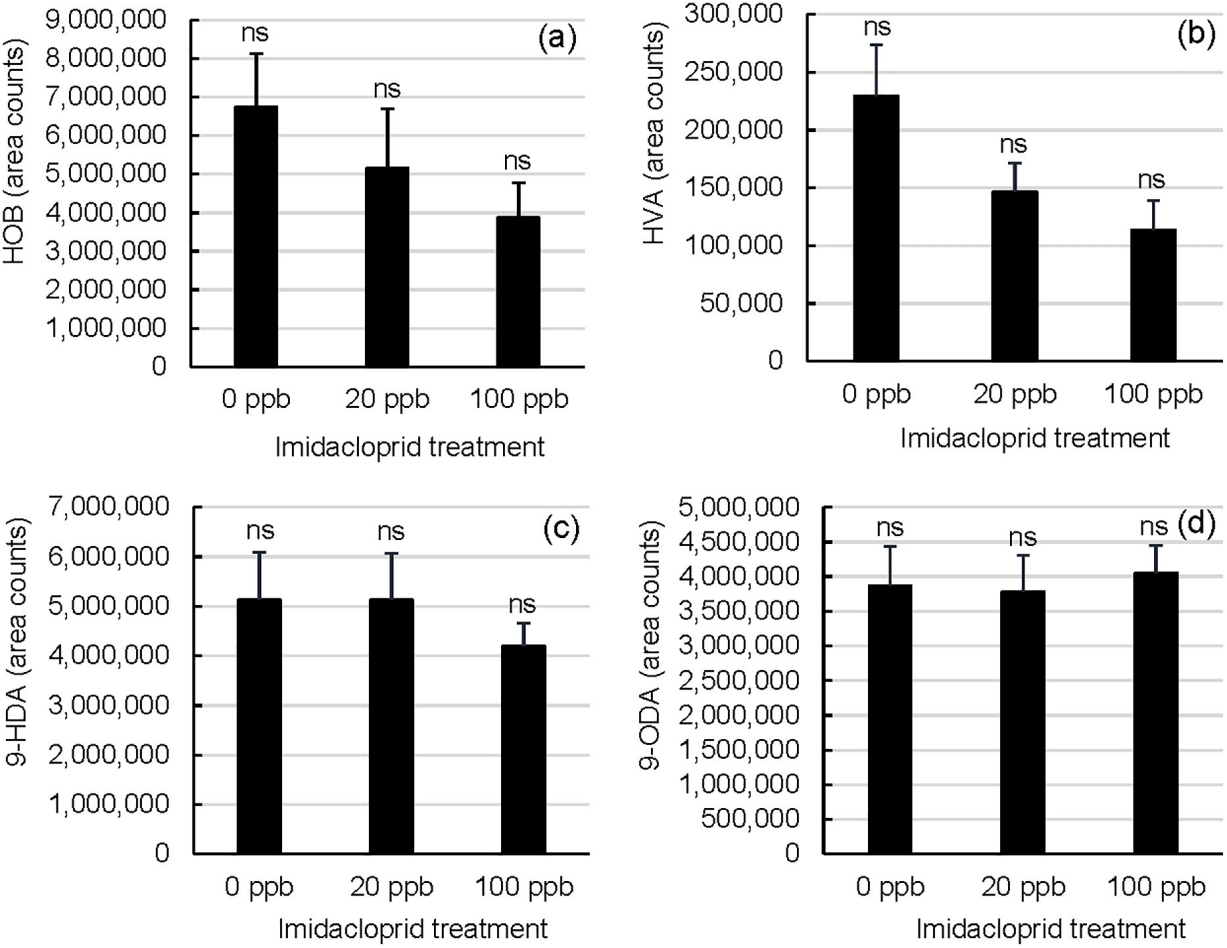
Relative QMP glandular component contents of winter queens from imidacloprid-supplemented and unsupplemented colonies. Queen QMP contents were compared as peak areas of characteristic m/z fragments for a given compound. The Queen Mandibular Pheromone (QMP) components a) methyl-p-hydroxybenzoate (HOB), b) 4-hydroxy-3-methoxyphenylethanol (HVA), c) 9-hydroxy-2-decenoic acid (9-HDA) and d) 9-oxo-2-decenoic acid (9-ODA) were extracted from queen mandibular glands. The two enantiomers of 9-HDA are reported together since these were largely indistiguishable. Treatment ranks were not significantly different by the Kruskal-Wallis test (p<0.05). Error bars represent S.E. (n = 12 (0 ppb imidacloprid in sugar syrup), n = 13 (20 ppb), or n = 10 (100 ppb)); treatment means that do not share a superscript differ by DSCF (queen ranks).

The four QMP compounds were reduced to two principal components PC1 and PC2 which explained 56.5% and 19.2% of the variance respectively (Fig 5). The proportions of these four QMP compounds did not significantly differ between treatment groups as indicated by comparisons across principal components (one-way ANOVA, (PROC ANOVA), F_2,32_ = 1.02, p = 0.3706 (PC1); F_2,32_ = 1.33, p = 0.2785 (PC2)). Imidacloprid treatments did not affect the individual queen glandular contents of any of the four major QMP components (Fig 6a–6d; ((HOB) Kruskal-Wallis test, Χ^2^ = 2.9544, df = 2, p = 0.2283; (HVA) Kruskal-Wallis test, Χ^2^ = 5.5856, df = 2, p = 0.0612; (9-HDA) Kruskal-Wallis test, Χ^2^ = 0.2360, df = 2, p = 0.8887; (9-ODA) Kruskal-Wallis test, Χ^2^ = 0.3620, df = 2, p = 0.8344). The queens, which appeared to receive similar levels of retinue worker care across treatments, contained similar amounts of each compound in their mandibular glands.

### Queen ovary and fat body soluble protein and lipid contents

**Fig 7:**
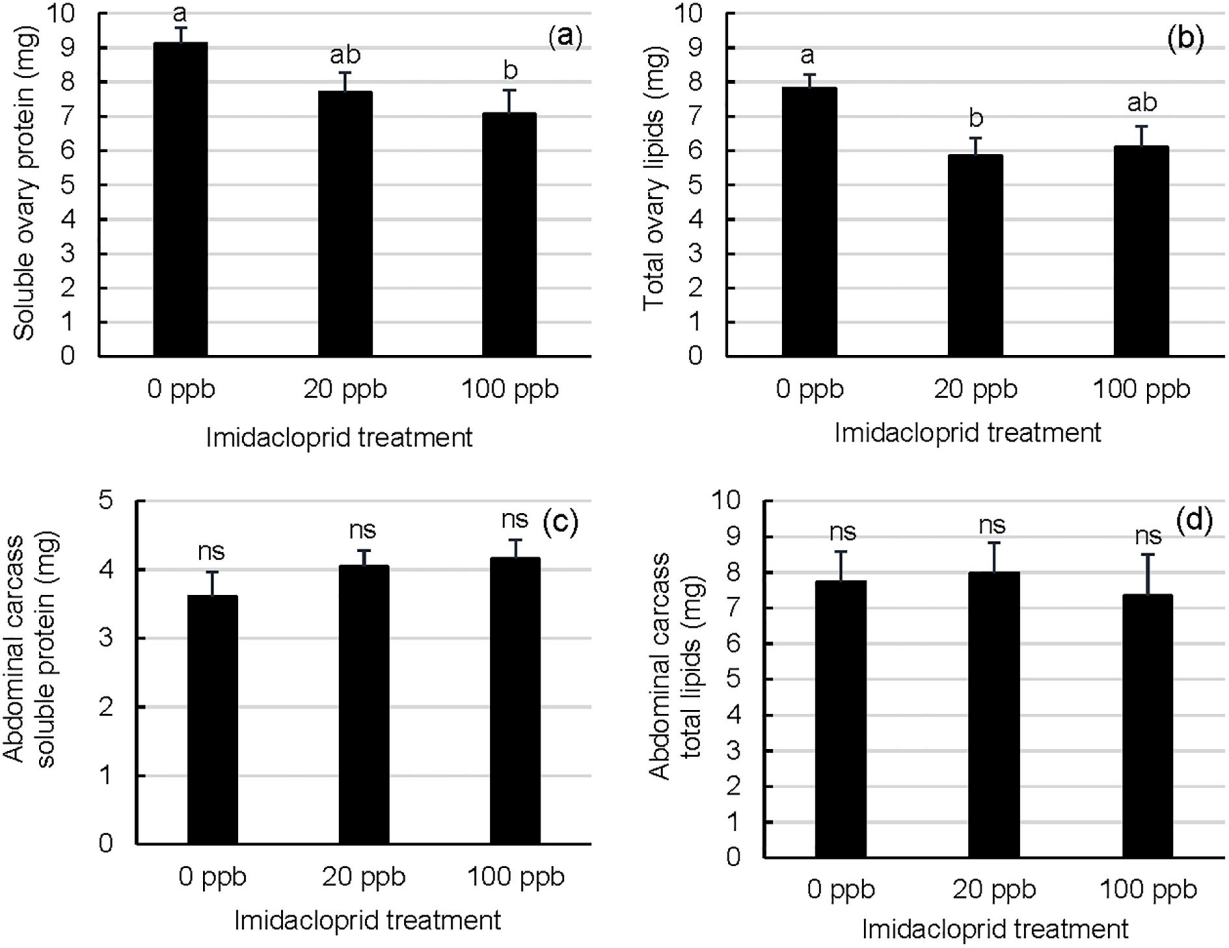
Reproductive and non-reproductive internal nutrient stores of winter queens from imidacloprid-supplemented and unsupplemented colonies. a) Total soluble protein and b) total lipid ovary contents and c) total soluble protein and d) total lipid fat body (abdominal carcass) contents were quantified from mid-winter queens. Sample queens were collected from colonies given sugar syrup supplemented with 0, 20, or 100 ppb imidacloprid. Error bars represent S.E. (n = 13 (0 ppb imidacloprid in sugar syrup), n = 14 (20 ppb), or n = 9 or 10 (100 ppb)); for a given metric, treatment means that do not share a superscript differ by Tukey’s HSD (queen means) or DSCF (queen ranks).

Exposure to high levels of imidacloprid moderately affected queen tissues critical to colony brood production. Mid-winter queens from 0 ppb colonies had marginally more soluble protein in their ovaries than queens from 100 ppb colonies and more lipids in their ovaries than queens from 20 ppb colonies (Fig 7a and 7b; Kruskal-Wallis test, Χ^2^ = 6.1376, df = 2, p = 0.0465 (protein); one-way ANOVA (PROC GLM) F_2,34_ = 4.51, df = 2, p = 0.0184 (lipids), DSCF (protein ranks) p<0.05; Tukey’s HSD (lipid means) p<0.05). By comparison, queen fat bodies (abdominal carcasses) did not differ by treatment group in total soluble protein or total lipid contents (Fig 7c and 7d; one-way ANOVA (PROC GLM) F_2,34_ = 0.95, p = 0.3977 (protein); one-way ANOVA (PROC GLM) F_2,34_ = 0.11, p = 0.8943 (lipids)).

## Discussion

Our experiment underscores the difference between being acutely exposed to neonicotinoid-contaminated nectar during foraging and chronically exposed through consumption of contaminated food stores. Paradoxically, honey bees extend their exposure to neonicotinoids by their ability to store and socially share contaminated food. In our experiment, imidacloprid residues persisted at high levels in nectar stores for at least 3 months after collection and storage. Sampled workers contained imidacloprid-contaminated stores at levels reflecting colony neonicotinoid contamination concentrations, although the presence of small amounts of imidacloprid residues in 0 ppb workers suggests limited exchanges between colonies by robbing. Other studies involving supplementation of colonies with imidacloprid-infused sugar syrup have recorded variable storage concentrations up to 160% of the original supplement concentration as dilute syrup is concentrated into honey [26, 38, 59]. The type of contaminated food material may determine the duration and extent of exposure among colony members. Unlike stored pollen, honey stores are rarely rapidly consumed and may be stored in comb for months before consumption. Initial worker exposure to and metabolic degradation of neonicotinoid residues in sugar stores likely occurs during collection and processing of contaminated nectar [58]. Once stored in honey cells, neonicotinoid residues remain relatively stable in a dark, acidic environment and persist for months [59]. Unlike more lipophilic insecticides, neonicotinoids partition into sugar solutions rather than comb wax while stored in honey cells. A critical element that is missing from our current assessment is how contact during foraging and food processing attenuates overall colony exposures to neonicotinoids (part of Wu-Smart’s and Spivak’s “dilution effects”) [38]. Many published studies like our own expose bees to neonicotinoid-contaminated pollen or nectar by placing supplements inside the colony [12, 38, 45, 47, 82]. Although this approach greatly reduces cross-contamination of non-target colonies, the use of in-hive treatments partially removes foraging and front-end food processing by worker physiology and enzymatic metabolism as factors in neonicotinoid degradation [33, 37, 58].

As in many previous studies, we detected sublethal effects of chronic imidacloprid exposure at very high doses (100 ppb) but less so at concentrations commonly associated with most crop applications (20 ppb and lower). Observed neonicotinoid concentrations in nectar or honey stores approaching 100 ppb are rare and largely restricted to guttation drops, certain ornamentals and to a lesser degree, certain specialty crops [13, 21, 22, 26, 38]. In our experiment, colonies exposed to lower concentrations of imidacloprid (20 ppb) suffered short term sublethal effects but appeared to recover or tolerate deleterious effects such that mid-winter colony performance was not significantly affected. Notably, reported impacts of chronic exposure to neonicotinoids on colony performance (i.e brood population, adult worker population, stored pollen accumulation) vary considerably between studies; furthermore, outcomes appear to be highly dependent on other colony stressors and the forage environment [12, 38, 47, 59, 83]. Chronic exposures to imidacloprid do not impact all colony functions equally, but especially target functions that require complex behavioral input or neural processing [29, 34–36]. The main impact of exposure to neonicotinoids may be on worker task efficiencies rather than effects on worker numbers alone. Exposure to imidacloprid appeared to reduce food collection and processing but not individual worker nutrition (as shown by HPG development). Similar impacts on foraging behaviors have been observed in other studies tracking individual workers with RFID tags. Neonicotinoids have been shown to reduce foraging efforts or efficiencies both in the short term (duration, ability to return to colony) and long term (onset of precocious foraging, lifetime foraging, and thus adult lifespan) [33, 65, 82]. These deleterious impacts on individual task efficiencies may have delayed effects on colony performance or may be compensated for over the long term. In our study, both brood production and adult worker populations were significantly reduced in mid-winter by exposure to 100 ppb imidacloprid syrup. Part of the impact of imidacloprid-contaminated sugar stores may depend on the degree to which age-related worker tasks expose bees to dietary neonicotinoids. Both brood rearing and foraging require consumption of large amounts of sugar stores for nursing and flight respectively [55].

Critically, the seasonal timing of exposure to neonicotinoids may affect a colony’s ability to tolerate other significant colony stressors such as overwintering. Our experiment differed from other recent field assessments of chronic neonicotinoid toxicity by the absence of a prolonged break between collection of neonicotinoid-contaminated food material and the stress of winter dearth. Other researchers have exposed colonies to dietary neonicotinoids in the summer to match characteristic unimodal patterns of seasonal floral abundance at their research sites [12, 38, 45, 47]. Colonies in these temperate locations may have had time and alternative floral resources to help recover from summer exposures to neonicotinoids before the onset of winter. By contrast, we exposed colonies to imidacloprid-contaminated syrup in the fall to simulate the bimodal seasonal abundance of forage in the lower Sonoran desert (late spring-early summer and late-summer-fall). Weeds and other uncultivated plants provide a significant part of forage outside of crop flowering and can readily be contaminated by neonicotinoid residues in soil and irrigation systems [21, 84]. This pattern of forage abundance with pronounced midsummer and early winter dearths is common in many warm subtropical climates except for Mediterranean climates [60]. As a result, our colonies were exposed to dietary imidacloprid during a period of seasonal stress and increased sugar consumption immediately preceding winter dearth. Honey bees build overwintering populations either by producing long-lived generalized workers (winter bees) or by continuing to produce short-lived replacement workers [85, 86]. Fall preparation for overwintering occurs at a time of increased dependence on honey stores as outside forage decreases. As winter approaches, adult workers including young nurse bees consume greater amounts of sugar stores to generate warmth for the colony [87]. Workers unable to maintain the hive temperature within the narrow range required by developing workers are less able to rear replacement workers of either type, eventually leading to rapidly shrinking adult populations [88, 89].

Our experiment did not extend through the overwintering and early spring expansion periods. In other long term studies on neonicotinoid toxicity, sublethal effects depended not only on dietary exposure and mode but also seasonal activities and other colony stressors. As a result, observed colony level impacts varied considerably and were sometimes delayed. Wood and coworkers (2019) found that colonies exposed to very high levels of thiamethoxam experienced reduced cluster sizes, lower sugar stores consumption, and reduced overwintering survival [90]. Sandrock and coworkers (2014) found short term effects on colony performance followed by long term increases in queen failures in the following spring [47]. Dively and coworkers noted that colonies given higher doses of imidacloprid (20 or 100 ppb) suffered no immediate effects on colony performance, but experienced delayed effects of queen failure and reduced brood rearing later in the summer and reduced overwintering survival [12]. Part of the variation in these impacts may be due to different susceptibilities of overwintering workers to dietary neonicotinoids. Neonicotinoids display greater toxicity to workers at colder temperatures experienced by overwintering colonies than in warmer conditions [91]. Individual overwintering workers are more susceptible to neonicotinoids than summer workers especially with bees near the spring brood expansion [92]. Yet unlike summer workers, overwintering workers preferentially consume sugar stores moderately contaminated by neonicotinoids [93]. Workers may become significantly less active after consuming highly contaminated food and may suffer reduced task efficiencies for critical worker tasks as a consequence [94].

It is not clear whether the observed effects of imidacloprid on colonies are due to direct impacts on queens or impacts on worker capabilities to perform essential queen and brood care behaviors. One stressor is less likely to be a cause of reduced brood rearing is severe worker malnutrition. The 100 ppb colonies showed a non-significant trend toward lower stored pollen accumulation during the fall forage periods. However, stored pollen was constantly available in most colonies from late summer to mid-winter. Furthermore, the young nest workers responsible for feeding other bees appeared to maintain well-developed HPG across treatment groups as indicated by total head protein. Workers produce jelly secretions with their HPG that form almost all of the food for ovipositing queens and young worker larvae, and part of the food for older larvae and other adult workers [64]. Nutritionally-stressed workers can possess substandard HPG that are less capable of feeding larvae either through poor development or premature gland degradation linked to accelerated adult maturation [74, 75]. An alternative explanation would be reduction of brood rearing activities by stressed colony workers. Honey bee workers often reduce population size during periods of stress and food shortages through brood cannibalism of eggs and young larvae [95]. These changes in worker brood rearing behaviors usually occur before food stores become completely depleted.

However, one factor that could readily result in reduced brood rearing are negative impacts on the resident queen. As the sole producer of female workers, even marginal reductions in queen oviposition rates during peak colony expansion periods (such as mid-fall) may result in notable population size differences. While we did not measure oviposition rates directly, neonicotinoid-exposed queens had slightly less protein and lipids in their ovaries than unexposed queens. Queen oviposition rates vary tremendously through the year ranging from complete cessation during extended dearths to over 1,800 eggs/day during peak colony expansion. While queens usually do not directly consume large amounts of honey stores, they may be indirectly exposed to neonicotinoids through consumption of trophallactic jellies. Retinue workers may feed queens with a few times their body mass in jelly secretions daily to produce colony eggs [54]. Queens from colonies fed lower concentrations of neonicotinoid contaminated treatment syrups may experience reduced oviposition rates in part due to slower movements [38]. Interpretation of our results is complicated by the marginal nature of continuous winter oviposition in warm subtropical and tropical locations. Queens in the Lower Sonoran desert may or may not substantially reduce ovipositing during winter depending in part on colony stressors such as forage scarcity and hard freezes [60, 62, 63, 73]. Admittedly, commonly used methods of assessing worker-queen interactions also may not fully capture worker behaviors that support queens. Although queen QMP gland contents and retinue size are commonly used to quantify queen signaling and worker attraction to queens, both methods rely on active responses by the involved bees. Workers support queen retention and oviposition through trophallactic feeding, grooming, and removal of contact QMP pheromones from the queen’s body to colony workers [67–70]. A salient feature of neonicotinoid impaired honey bees is reduced movements and activity levels. Wu-Smart and Spivak noted that imidacloprid-exposed queens move slower and oviposit less often than unexposed queens [38]. While we did not systematically assess worker task behaviors in this study, we generally did not observe gross impairment of basic worker tasks essential to colony maintenance. Neonicotinoids are thought to interfere progressively with worker behaviors with increasing exposure [96]. While somewhat listless, even workers from 100 ppb colonies engaged in expected warm subtropical overwintering behaviors including trophallactic feeding of brood, workers and queens as well as hygienic behaviors, food consumption, guarding, and warming of the hive interior during cooler periods [62, 97]. A more likely mode is sublethal impairment of worker tasks activities, learning, and responses to external stimuli.

Fortunately, deleterious effects of imidacloprid from contaminated nectar stores may be partially mitigated by providing uncontaminated food alternatives. Our results suggest that the impact of high levels of imidacloprid on honey bees depends on factors not only including dosage but also the timing of exposure and the availability of alternative sugar sources. Neonicotinoid contents of honey cells may determine how workers consume contaminated materials [93]. Worker bees have been shown to prefer sugar solutions with low concentrations of neonicotinoids but also avoid sugar solutions containing high concentrations of neonicotonoids [12, 29, 93, 98]. Colonies with access to alternative sugar sources might reduce the effective concentration of ingested nectar stores by dilution with supplemental sugar or outside floral resources (part of Wu-Smart’s and Spivak’s “dilution effect”) [38]. By this concept, the collection of uncontaminated nectar or supplemental sugar syrup would dilute the effective concentration of neonicotinoid-contaminated sugar stores consumed by the colony. Importantly, our observed sublethal effects and overwintering colony losses primarily occurred at 100 ppb imidacloprid levels exceeding most field-realistic field exposures (<10 ppb) and only marginally at neonicotinoid levels associated with very high field exposures (20 ppb) [12, 13, 16, 38]. Future studies may consider management practices designed to provide uncontaminated food alternatives (i.e. uncontaminated honey frames, feeding supplemental sugar in dearth, providing supplemental forage in dearth) that effectively dilute contaminated food stocks. Both managed and native pollinators face declines in natural forage outside of crop systems worldwide due to expanded agricultural production and more intensive management of field margins [1, 2]. An emphasis on targeted alternatives outside treated crop systems may lessen impacts of neonicotinoids on honey bee colonies at their most vulnerable time of the year.

## Supporting information

S1 Fig Head protein contents of pooled nurse workers from imidacloprid-supplemented and unsupplemented colonies. Error bars are S.E. (pooled samples by colony, n = 17 (0 ppb imidacloprid in sugar syrup), n = 16 (20 ppb), or n = 15 (100 ppb, first to last time point)). Treatment means were not significantly different within each time point by Tukey’s HSD (colony means).
(TIF)

S2 Fig Number of sperm stored in queen spermatheca in winter queens from imidacloprid-supplemented and unsupplemented colonies. Error bars are S.E. Treatment means were not significantly different by Tukey’s HSD (queen means, n = 12 (0 ppb), 14 (20 ppb), and 11 (100 ppb)).
(TIF)

S1 File All data sets except RFID. (XLSX)

S2 File RFID data set. (XLSX)
