# Supplementary figures and images for: Sublethal effects of imidacloprid-contaminated honey stores on colony performance, queens, and worker activities in fall and early winter colonies
Source: PLoS One. 2024 Jan 2;19(1):e0292376. doi: 10.1371/journal.pone.0292376 (PMC10760783; doi:10.1371/journal.pone.0292376)

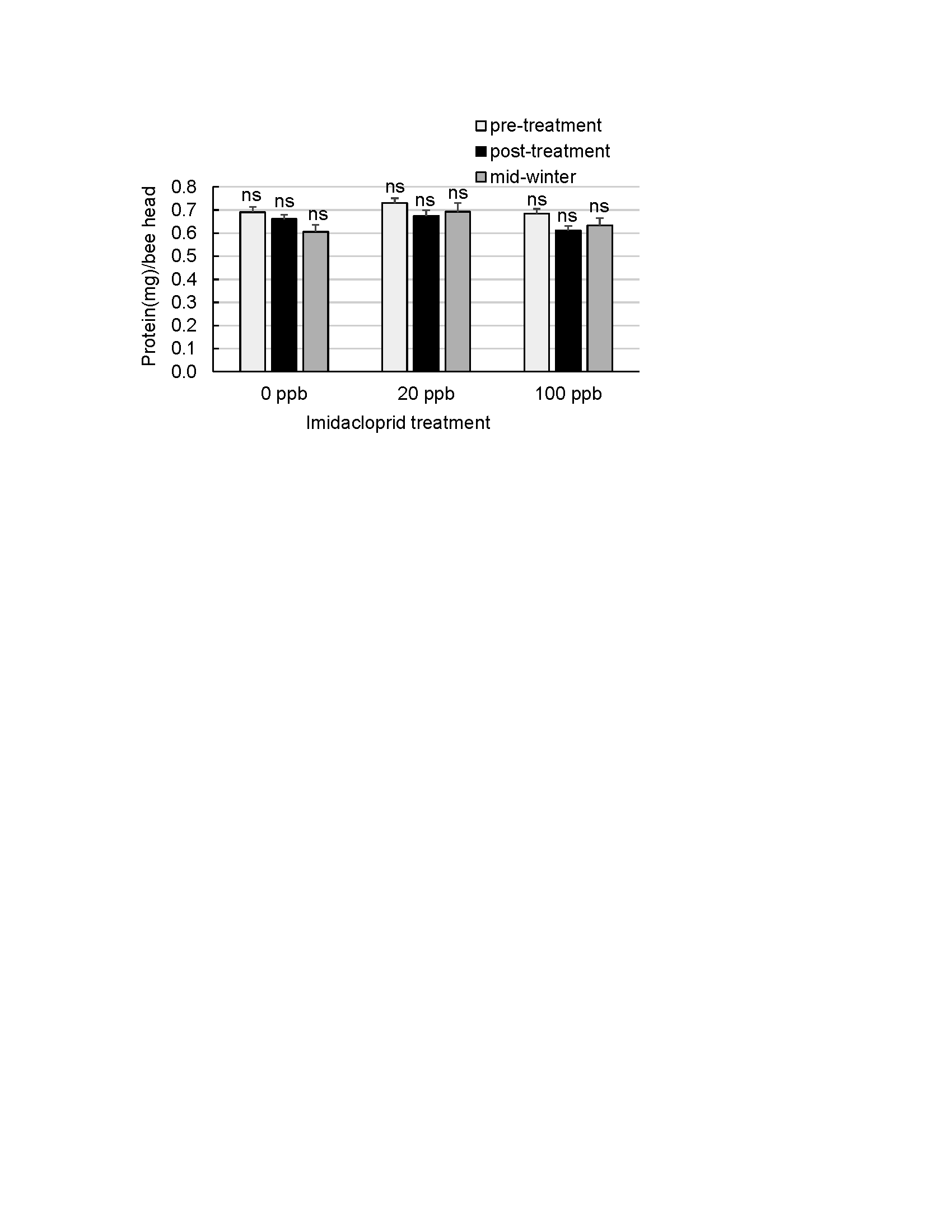

Supplement: S1 Fig — Error bars are S.E. (pooled samples by colony, n = 17 (0 ppb imidacloprid in sugar syrup), n = 16 (20 ppb), or n = 15 (100 ppb, first to last time point)). Treatment means were not significantly different within each time point by Tukey’s HSD (colony means). (TIF) [file pone.0292376.s001.tif]

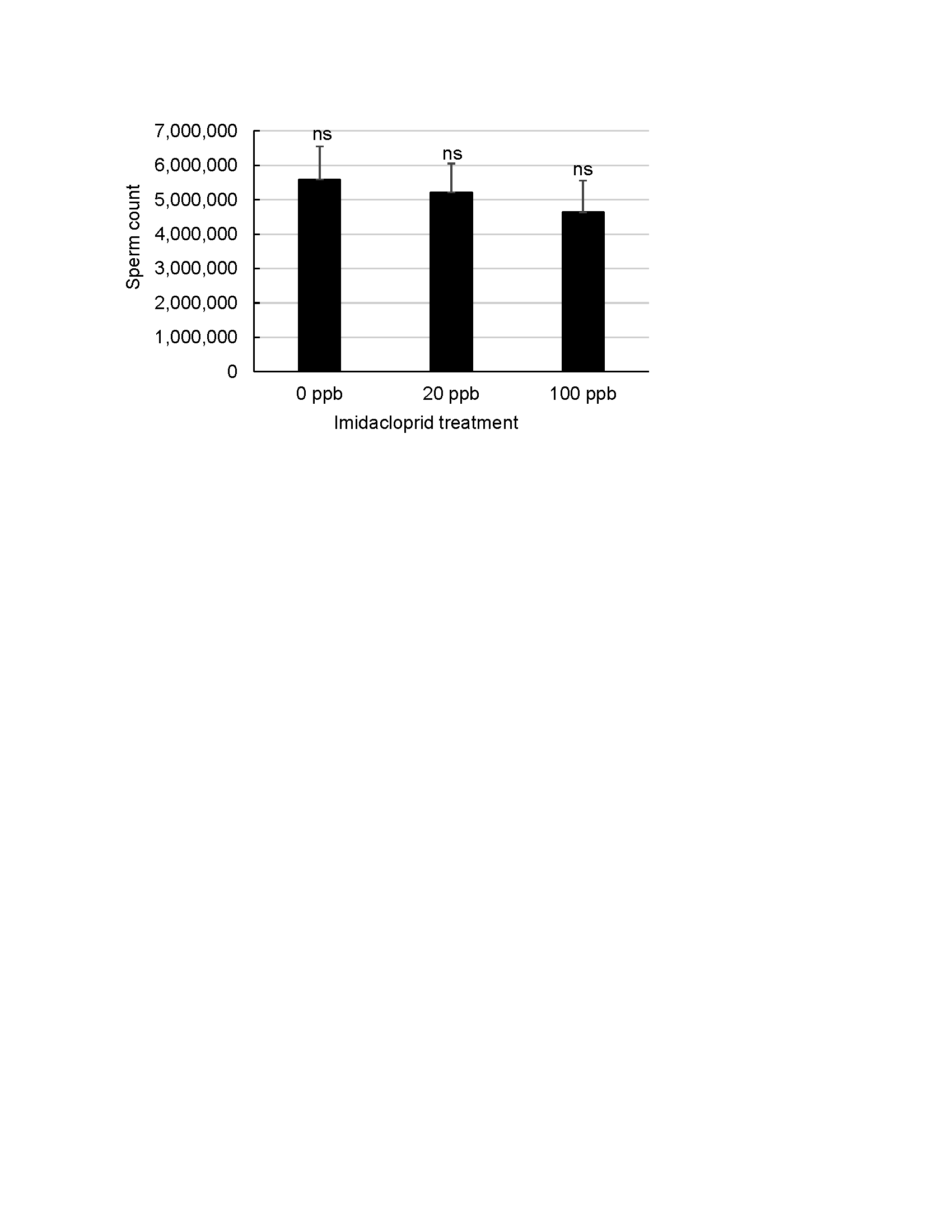

Supplement: S2 Fig — Error bars are S.E. Treatment means were not significantly different by Tukey’s HSD (queen means, n = 12 (0 ppb), 14 (20 ppb), and 11 (100 ppb)). (TIF) [file pone.0292376.s002.tif]
